# Conservation of the Keap1-Nrf2 System: An Evolutionary Journey through Stressful Space and Time

**DOI:** 10.3390/molecules22030436

**Published:** 2017-03-09

**Authors:** Yuji Fuse, Makoto Kobayashi

**Affiliations:** 1 Department of Molecular and Developmental Biology, Faculty of Medicine, University of Tsukuba, Tsukuba 305-8575, Japan; fuse_yuji@md.tsukuba.ac.jp; 2 Doctoral Program in Biomedical Sciences, Graduate School of Comprehensive Human Sciences, University of Tsukuba, Tsukuba 305-8575, Japan

**Keywords:** anti-stress system, *Drosophila* Cnc, evolutionary history, *Hydra* Nrf, Keap1, mouse, Nrf2, *C. elegans* Skn-1, yeast Yap1, zebrafish

## Abstract

The Keap1-Nrf2 system is an evolutionarily conserved defense mechanism against oxidative and xenobiotic stress. Its regulatory mechanisms, e.g., stress-sensing mechanism, proteasome-based regulation of Nrf2 activity and selection of target genes, have been elucidated mainly in mammals. In addition, emerging model animals, such as zebrafish, fruit fly and *Caenorhabditis elegans*, have been shown to have similar anti-stress systems to mammals, suggesting that analogous defense systems are widely conserved throughout the animal kingdom. Experimental evidence in lower animals provides important information beyond mere laboratory-confined utility, such as regarding how these systems transformed during evolution, which may help characterize the mammalian system in greater detail. Recent advances in genome projects of both model and non-model animals have provided a great deal of useful information toward this end. We herein review the research on Keap1-Nrf2 and its analogous systems in both mammals and lower model animals. In addition, by comparing the amino acid sequences of Nrf2 and Keap1 proteins from various species, we can deduce the evolutionary history of the anti-stress system. This combinatorial approach using both experimental and genetic data will suggest perspectives of approach for researchers studying the stress response.

## 1. Introduction

From birth, animals are destined to fight against a variety of stressors that disrupt their homeostasis. All animal species must cope with oxidative stress generated by their own metabolism. They were also forced to evolve detoxifying systems in case of accidental encounters with toxic chemicals in the environment. Animals could not have prospered without the anti-stress mechanisms evolved by their ancestral species.

Mammals inherited from their ancestors the Kelch-like ECH-associated protein 1 (Keap1)-NF-E2-related factor 2 (Nrf2) system, which is a defense system that confers protection against a wide spectrum of stressors, including oxidative and chemical stress. Recent advances have revealed that the system is related to a number of human diseases, such as cancer, neurodegenerative diseases and diabetes mellitus [1,2,3], and many researchers are investigating potential medical applications. Although the Keap1-Nrf2 system has mainly been studied using mice and human cells, increasing evidence suggests that orthologous systems exist in lower vertebrates, such as zebrafish [4], and even in *Drosophila* [5], which will be promising tools for accelerating the study of Nrf2.

The advantages of lower-model animals are not limited to their usefulness in the laboratory and few ethical problems. Recent improvements in genome projects have provided high-quality genomic sequences, facilitating the development of lower-model organisms and enabling us to assess specific biological functions from an evolutionary point of view. In addition to classic model animals, genomic information from non-model animal species has been increasingly accumulated. This information prompted us to track the evolutionary path of proteins of interest.

In the present review, we will highlight the evolution of the Keap1-Nrf2 system and attempt to identify the unique characteristics of the mammalian Nrf2 system by reviewing the evolutionary history of the conserved anti-stress mechanism.

## 2. Overview of the Keap1-Nrf2 System

### 2.1. Transcription Factor Nrf2 and Its Function

Nrf2 was discovered as a homolog of nuclear factor-erythroid 2 p45 (NF-E2), which plays an essential role in the transcriptional regulation of the β-globin gene [6,7]. Unlike NF-E2, the function of this newly-discovered transcription factor was not related to hematopoiesis; Nrf2-knockout mice did not show any obvious phenotype and were normally grown and fertile with no anemia [8,9,10], suggesting that Nrf2 regulates a different battery of genes from NF-E2.

The function of Nrf2 was first reported by Itoh et al. [11], who noticed the similarity between the NF-E2 binding sequence and antioxidant responsive element (ARE). This regulatory sequence is usually found upstream of genes encoding phase II detoxifying enzymes and had been known to regulate the induction of these genes [12,13]. The down-regulation of the expression of phase II enzymes in Nrf2-knockout mice indicated that Nrf2 regulated the global transcription of phase II enzymes through ARE-dependent signals [11].

The main function of phase II enzymes is to detoxify the highly reactive intermediate metabolites generated by phase I reactions and accelerate the excretion of toxic xenobiotics [14]. Benzo[*a*]pyrene is a well-studied pro-carcinogen that forms a highly reactive intermediate after phase I metabolism and is detoxified by phase II reactions. The loss of Nrf2 was suspected to potentially weaken phase II metabolism and enhance the carcinogenicity of benzo[*a*]pyrene. This was experimentally demonstrated by showing the high susceptibility of Nrf2-deficient mice to benzo[*a*]pyrene-induced tumor formation, suggesting that Nrf2 is indispensable for intact phase II metabolism [15,16,17]. Later studies revealed that the Nrf2 system also regulates phase III xenobiotic transporters [18,19] as well as phase I-related genes [20,21], suggesting that Nrf2 is involved in the entire process of xenobiotic metabolism. In addition, as ARE sequences were also found upstream of antioxidative genes, such as heme oxygenase 1, Nrf2 was shown to be the master regulator of the oxidative stress response [22]. The mode of action of many toxic chemical stressors is the generation of reactive oxygen species; therefore, Nrf2 plays an important role in the defense against various chemical-derived stresses, such as diesel exhaust, peroxide, heavy metals and other electrophilic compounds [23,24,25]. The Nrf2-dependent induction of xenobiotic metabolism and antioxidant system likely contributed greatly to animal evolution in a rapidly changing environment.

The Nrf2 system is also activated by endogenous cues, such as endoplasmic reticulum (ER) stress, and confers defense against such stress [26,27]. This protection may be partly due to the Nrf2-dependent induction of proteasome subunits [28,29], which destroy unfolded proteins accumulated in the cell. Another endogenous Nrf2-activating signal is the disruption of autophagy [30], which implies that the Nrf2 system can be activated by disorders of protein turnover. Numerous studies have shown that these internal stresses are related to various diseases; as such, Nrf2 has attracted attention as a potential therapeutic target.

By contrast to these beneficial effects on human health, adverse effects of the Nrf2 system have also been reported: Nrf2 is constitutively activated in cancer cells and confers resistance against chemotherapy [31,32,33,34]. Surprisingly, Nrf2 activates the pentose phosphate pathway and remodels cellular metabolism, which enhances cancer cell proliferation [35,36]. These findings may support the advent of Nrf2-blocking therapy for cancer patients.

### 2.2. Regulatory Mechanism of Nrf2-Dependent Gene Induction

**Figure 1:**
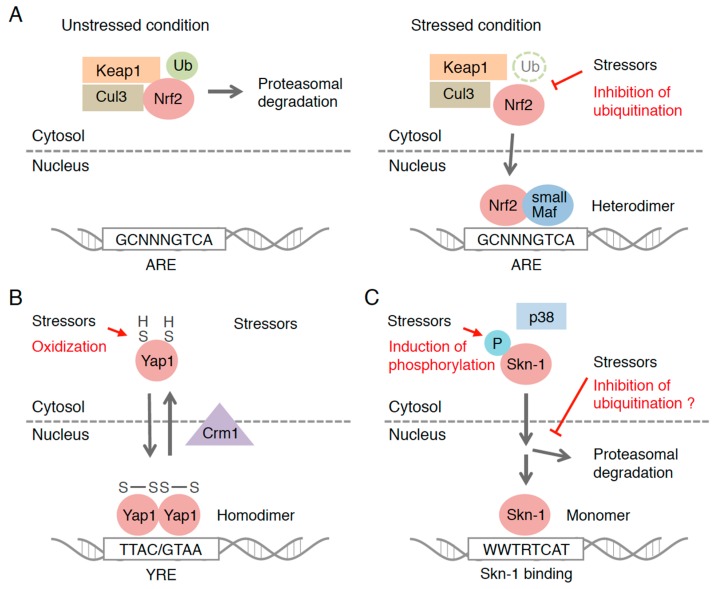
Regulatory mechanisms of the transcription factor-based oxidative stress response in eukaryotes. The activation mechanism of: Keap1-Nrf2/Cnc system (**A**); Yap1 in *S*. *cerevisiae* (**B**); and Skn-1 system in *C. elegans* (**C**) are depicted.

Research on the Nrf2 activation mechanism has greatly progressed since a partner protein of Nrf2, Keap1, was discovered (Figure 1A). Keap1 was discovered as a protein that binds directly to Nrf2 and negatively regulates the transcriptional activity of Nrf2 [37]. As Keap1 is the adaptor protein for the ubiquitin ligase, Nrf2 is ubiquitinated in a Keap1-dependent manner and degraded by proteasome system [38,39,40], which keeps the Nrf2 protein level low under unstressed conditions. Nrf2-activating compounds inhibit the function of Keap1 by attacking highly reactive cysteine residues and stabilize Keap1-Nrf2 binding. De novo-synthesized and accumulated Nrf2 translocates into the nucleus and binds to ARE with its heterodimeric partner, small musculoaponeurotic fibrosarcoma (small Mafs: MafG, MafK and MafF), to induce the target genes [7,41,42,43,44]. Since Keap1 receives the redox information or environmental cues via its highly reactive cysteine residues, it is often called the “sensor” molecule that determines the responsiveness of Nrf2-dependent gene induction.

In addition to the Keap1-dependent degradation, Nrf2 protein is also regulated in a Keap1-independent manner. Salazar et al. [45] revealed that glycogen synthase kinase-3β (GSK-3β) inhibits Nrf2 activity by direct phosphorylation. Phosphorylated Nrf2 then interacts with β-transducin repeat-containing protein (β-TrCP), a substrate receptor for ubiquitin ligase complex, and is ubiquitinated [46]. Although the physiological context in which this phosphorylation pathway is modulated is unclear, Chowdhry et al. [47] reported that the inhibited GSK-3 pathway in cancer cells activates Nrf2 and confers drug resistance by upregulating anti-stress genes.

These regulatory mechanisms of the Keap1-Nrf2 system have been identified mainly using mammalian cells. Non-mammalian animals also have anti-stress systems analogous to the mammalian Keap1-Nrf2 system. In the next section, we will overview these analogous systems in lower animals.

## 3. Overview of the Anti-Stress Systems in Lower Model Organisms

### 3.1. Yap in *Saccharomyces cerevisiae*

Studies of budding yeast, *Saccharomyces cerevisiae*, have shown that the transcription-based stress response stems from single-cell eukaryotes. Yap family proteins are a well-studied group of transcription factors that confer protection against oxidative and chemical stress [48]. The Yap family consists of eight paralogs of basic leucine zipper (bZip)-type transcription factors, Yap1-8. Of these members, Yap1 is the major isoform that confers protection against oxidative stress (Figure 1B) [49]. Unlike Nrf2 in mammals, Yap1 forms homodimers that bind to specific sequences of DNA, Yap response element (YRE), and activate the transcription of target genes. The stress-sensing system is also unique. Yap1 has cysteine residues in its C-terminus that function as sensors. Under unstressed (reduced) conditions, Yap1 localizes in the cytosol by the action of exportin chromosomal maintenance 1 (Crm1), and the transcriptional activation is inhibited. When exposed to oxidative stress, the cysteine residues of Yap1, however, are oxidized with the assistance of glutathione peroxidase 3 (Gpx3), a thiol peroxidase, and an intramolecular disulfide bond is formed [50,51,52,53]. In this structure, Crm1 cannot approach the nuclear export signal (NES) region of Yap1, resulting in the nuclear retention of Yap1 and its target gene activation. Regarding this unique activation mechanism, the Yap1 system might stem from a different evolutionary origin from the Keap1-Nrf2 system.

### 3.2. Cnc in *Drosophila*

The fruit fly, *Drosophila melanogaster*, is a classic model animal with substantial advantages in the fields of developmental biology and genetics. The fly homolog of Nrf2 was discovered as an important protein in the development of the cranial portion (labral and mandibular structure) of larvae [54]. Because of its unique expression pattern, this gene was named Cap’n’collar (CNC) [55]. Despite highly conserved amino acid sequences with Nrf2, the anti-stress function of Cnc was not described until the discovery of the transcript variant, CncC, which contains N-terminal domains homologous to Nrf2 [56,57]. CncC was demonstrated to have an anti-stress function in adult flies [58]. In addition, the activity of CncC was regulated at the protein level by the direct interaction with Keap1 [58,59], and heterodimerization with *Drosophila* small Maf protein, Maf-S, was also demonstrated [60]. The target genes of fly CncC are similar to those of Nrf2 in mammals. Phase I and II enzymes, antioxidant proteins and proteasome subunits are shown to be under the regulation of CncC [58,61,62,63]. These analogies suggest that the Keap1-CncC system in *Drosophila* evolved from a common ancestral system with the mammalian Keap1-Nrf2 system.

### 3.3. Skn-1 in *Caenorhabditis elegans*

The laboratory worm *Caenorhabditis elegans* is also a strong model in developmental biology, and skinhead-1 (Skn-1), an ortholog of Nrf2 in mammals, was discovered as a protein that is important for normal pharyngeal development [64]. Skn-1 has a similar inducible defense function to Nrf2, namely protection against chemical stresses [65]. Uniquely, Skn-1 binds to specific DNA sequences as a monomer due to the loss of leucine zipper domain, which is important for dimerization with small Mafs in mammals (Figure 1C) [66]. Although the transcriptional activity of Skn-1 seems to be regulated at the protein level, *C*. *elegans* does not have an authentic ortholog of Keap1 [67]. Instead, in *C*. *elegans*, the WD40 repeat protein-23 (WDR-23)/damaged DNA binding protein 1 (DDB1) complex is involved in the ubiquitination of Skn-1 under basal conditions [68]. However, it remains unclear how Skn-1 escapes this negative regulation in stressed situations. Phosphorylation-based regulation has also been described in the Skn-1 system, and three kinases—AKT, PMK-1 (p38) and GSK-3—were shown to target the serine residues on Skn-1 [69,70,71]. Of these kinases, phosphorylation by PMK-1 activates Skn-1, while the others negatively regulate the Skn-1 function. Mammalian Gsk-3 and p38 are also known to be involved in Nrf2 regulation [45,72]; therefore, this phosphorylation-based regulation may be conserved from an ancestral system.

Although the regulatory mechanism differs from that of the mammal Nrf2 system, the target genes of Skn-1 are similar to those of mammalian Nrf2. The gene expressions of phase I, II and III detoxifying enzymes, antioxidant proteins and proteasome subunits are regulated in a Skn-1-dependent manner [65,68,73]. This implies that Keap1-Nrf2/CncC and Skn-1 stem from the same ancestral system, while in *C*. *elegans*, a unique regulatory mechanism was evolved.

### 3.4. Nrf2 in Zebrafish

In zebrafish (*Danio rerio*), an emerging model animal in medical research, the Keap1-Nrf2 system is highly conserved with that of the mammalian system. Zebrafish Nrf2 and Keap1 were first cloned in 2002 and found to be structurally similar proteins to their mammalian counterparts [57]. Dimeric partners of zebrafish Nrf2 have also been identified and revealed to have conserved small Mafs, MafG (co-ortholog MafG1 and MafG2) and MafK, along with fish-specific MafT (a possible ortholog of mammalian MafF). All of these homologs can function as binding partners of zebrafish Nrf2 [74]. The function of the upstream ARE sequence was shown to be necessary for the Nrf2-dependent induction of a gene encoding phase II enzyme, *gstp1* [75]. The defense function against xenobiotics and oxidative stress was also demonstrated in vivo using Nrf2 mutant zebrafish strain [76,77]. Lineups of Nrf2 target genes are also conserved in zebrafish. Proteins involved in the detoxification pathway, antioxidant proteins, proteasome subunits and pentose phosphate pathway enzymes are also regulated by the Nrf2 system in zebrafish [77,78,79,80]. This experimental evidence clearly shows that vertebrates have an evolutionarily conserved Keap1-Nrf2 system.

## 4. Evolution of Nrf Protein

### 4.1. Comparison of Nrf Protein Structures

The existence of an analogous Keap1-Nrf2 system in *Drosophila* suggests that this system is conserved among a wide range of species throughout the animal kingdom. We attempted to trace the evolutionary path of the Keap1-Nrf2 system using genomic information from various animal species (Table 1). In addition to laboratory animals, including mice, zebrafish, *Drosophila* and *C*. *elegans*, the predicted protein sequences of Nrf were obtained from ascidians (*Ciona intestinalis*) [81], sea urchin (*Strongylocentrotus purpuratus*) [82], octopus (*Octopus bimaculoides*) [83] and the diploblastic metazoan *Hydra magnipapillata* [84].

**Figure 2:**
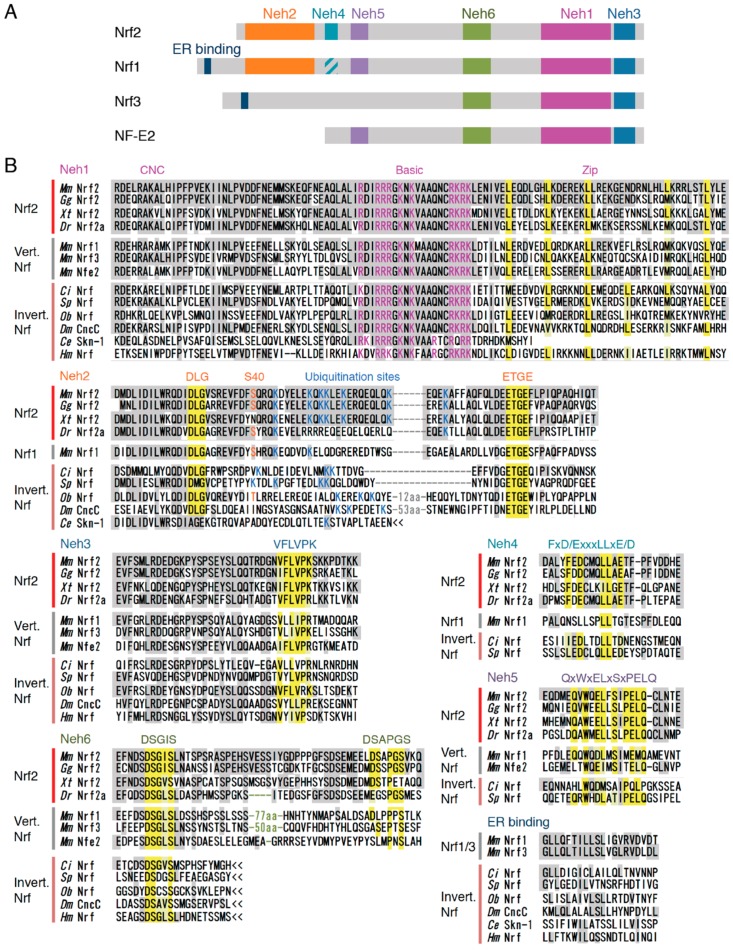
Comparison of Nrf family proteins. (**A**) The domain structures of four Nrf proteins in mammals were compared based on the six Neh domains of Nrf2. Striped color denotes the region that is partially conserved with the Neh4 domain. The ER binding domain (NHB1) that is specific to Nrf1 and -3 is also indicated; (**B**) Amino acid sequences of Nrf/Cnc transcription factors from mouse (*Mm*), chicken (*Gg*), clawed frog (*Xt*), zebrafish (*Dr*), ascidian (*Ci*), sea urchin (*Sp*), octopus (*Ob*), fruit fly (*Dm*), *C*. *elegans* (*Ce*) and *Hydra* (*Hm*). The amino acids identical to mouse Nrf2 are shaded in gray. Leucine residues comprising the zipper structure in Neh1, the DLG and ETGE motifs in Neh2, the VFLVPK motif in Neh3, the FxD/ExxxLLxE/D sequence in Neh4, the QxWxELxSxPELQ sequence in Neh5 and the DSGIS and DSAPGS motifs in Neh6 are shaded in yellow. Basic amino acid residues in the Neh1 basic region, lysine residues between the DLG and ETGE motif (ubiquitination sites) and serine/threonine residues (Ser-40, phosphorylation site) in the Neh2 domain are shown in pink, blue and orange letters, respectively.

Vertebrates have 4 Nrf genes—Nrf1, Nrf2, Nrf3 and NF-E2 (Figure 2A)—while lower animals (ascidians, sea urchin, octopus, fly and *Hydra*) seem to have only 1 Nrf gene locus, implying the diversification of this protein family in vertebrate evolution. Of the Nrf family proteins in mammals, Nrf1 has the most similar domain structure to Nrf2 and is known to regulate the transcription of antioxidant and phase II enzymes through binding to ARE, forming heterodimers with small Mafs. Despite this similarity to Nrf2, the regulatory mechanism of Nrf1 differs substantially from that of the Nrf2 system, largely due to the localization to the ER membrane by its ER binding region in the N-terminal domain, while Nrf2 stays in the cytosol under unstressed conditions. In addition, activity of Nrf1 is regulated by the glycosylation/deglycosylation, not by a Keap1-dependent ubiquitination mechanism [85]. Similarly, Nrf3 is tethered to the ER membrane by its N-terminal region, and heterodimerization with small Mafs is needed for DNA binding [86,87]. Studies of the Nrf3 function are still in progress [88]. NF-E2 is specifically expressed in hematopoietic tissue and also need to make heterodimer with small Mafs for the transcriptional regulation of target genes [89,90]. Genetic studies have shown that NF-E2-knockout mice scarcely survived after the neonatal stage because of the absence of platelets and severe hemorrhaging [91], and genetic ablation of Nrf1 resulted in embryonic lethality [92,93]. In contrast, mice with Nrf2 and Nrf3 knockout were viable and fertile [8,11,94], suggesting that the functions of these four Nrf proteins have significantly diverged during vertebrate evolution. Below, we compared the amino acid sequences of the Nrf proteins from various species and four Nrf family proteins in mice (Figure 2B). Based on the alignment, the specific features of each protein were extracted.

The Neh1 domain, also known as the CNC-bZip domain, is an essential region for DNA binding (CNC domain and basic region) and dimerization with small Maf proteins (leucine zipper region). The amino acid sequence of this region, especially the basic region in the middle of this domain, is highly conserved among a wide range of species, suggesting that all these proteins were derived from the same origin, and that the DNA binding ability has been inherited from ancestral species. Interestingly, Skn-1 was observed to have lost the leucine zipper, an essential region for the dimerization, which may therefore explain why Skn-1 binds to DNA as a monomer.

The Neh2 domain is characterized by its interaction with Keap1, making it an essential domain for the proteasome-dependent degradation of Nrf2 [37]. Direct interactions of two motifs (DLG at the N-terminus and ETGE at the C-terminus) with Keap1 are necessary for the normal turnover of Nrf2 protein [57,95,96,97]. The Neh2 domain was not found in the *Hydra* Nrf protein, but Nrf of other triploblastic animals all have this domain. Nrf1 also possess these two motifs, while NF-E2 and Nrf3 have lost the Neh2 regions. Another important point is the conservation of lysine residues between the DLG and ETGE motifs, as seven lysine residues in this region of mouse Nrf2 were shown to be ubiquitinated, which promotes proteasomal degradation [98]. Although the number of lysine residues varies among species, all Nrf2 proteins possess at least two in this area, suggesting the conservation of Nrf2 degradation through the ubiquitin proteasome system. Another important amino acid is Ser-40, which is phosphorylated by protein kinase C. Phosphorylation of this serine has been suggested to promote dissociation from Keap1 [99]. This site has only been observed in vertebrates, except for some animals such as *Xenopus tropicalis*, fugu, medaka fish and platypus, suggesting that the regulation of the Keap1 binding affinity by phosphorylation evolved after the appearance of vertebrates, but it was subsequently lost in some species.

The C-terminal Neh3 domain was shown to be crucial for the transactivation of target genes. Mutant Nrf2 proteins with an incomplete Neh3 domain cannot activate ARE-dependent gene expression [100]. In particular, the VFLVPK motif in this region interacts with chromodomain helicase DNA binding protein 6 (CHD6), which may be indispensable for the full activity of Nrf2. The amino acid sequence of this motif was also highly conserved among all of the animals investigated.

The Neh4 and Neh5 domains are also transcriptional activation domains, which interact with transcriptional co-activator CREB binding protein (CBP). Transcriptional adapter motif (TRAM) (FxD/ExxxL) in Neh4 is considered to be an essential motif for this interaction [101]. No homologous region of the Neh4 or Neh5 domains was found in *Hydra* or in protostomes, *Drosophila* or octopus Nrf, implying that these domains evolved only in deuterostomes. In sea urchins and ascidians, conserved Neh4 and -5 domains were found. Teleost Nrf2 proteins (amazon molly, cavefish, cod, fugu, medaka, platyfish, spotted gar, stickleback, tetraodon, tilapia and zebrafish) have complete TRAM as well as a conserved Neh5 domain, although Nrf2a in cavefish does not have a conserved Neh5 domain (http:www.ensembl.org). Regarding other Nrf family proteins in mice, TRAM was incomplete in Nrf3 and was not found in Nrf1 or NF-E2. The Neh5 domain was conserved in Nrf1 and NF-E2 but not in Nrf3.

The Neh6 domain plays a role in the phosphorylation-based regulation of Nrf2 activity. The DSGIS motif in the N-terminus of Neh6 is essential for the GSK-3β-dependent phosphorylation and subsequent ubiquitination by β-TrCP [47]. This motif was found to be conserved in *Hydra*; therefore, the Nrf activity might be regulated by phosphorylation-dependent degradation from common ancestors of species investigated. This motif was also conserved in other Nrf proteins in mice, suggesting that the activity of these isoforms is also modulated by phosphorylation. The Neh6 domain of mouse Nrf2 has another phosphorylation site in its C-terminus (DSAPGS motif) [47], but no conserved amino acid sequence was observed in Nrf of *Hydra*, fly, octopus, sea urchin or ascidian. Although this motif was found in vertebrate Nrf proteins, the conservation of the amino acid sequence was relatively weak, and the functional importance of this region is unclear.

We next searched for homologous sequences to the N-terminal ER binding region of mouse Nrf1 and its vertebrate orthologs. N-terminal homology box1 (NHB1) was shown to be important for Nrf1 tethering to the ER [102]. Nrf3 of mouse, frog and zebrafish have a highly conserved NHB1 region to Nrf1. The Nrf proteins of lower animals also have a homologous region, and the N-terminal half of this region in particular tends to be highly conserved.

**Figure 3:**
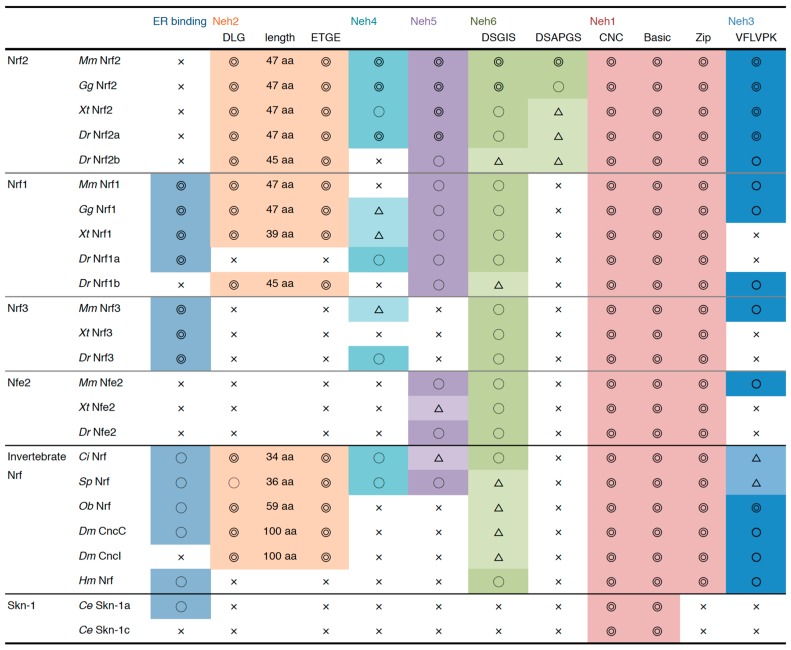
A summary of the domain structure of the Nrf/Cnc transcription factors. Conservation of the Neh domains was evaluated as follows: ◎, highly conserved; ○, relatively conserved; △, partially conserved; ×, not conserved. Specific motifs were described as “highly conserved” only when the sequences were identical to mouse Nrf2. The amino acid lengths between DLG and ETGE motifs are also shown.

The conservation of each domain is summarized in Figure 3. Of the diversified Nrf proteins, only vertebrate Nrf2 has all six Neh domains. In particular, TRAM in the Neh4 domain was well conserved in the Nrf2 isoform, while it was unclear in Nrf1 and Nrf3. Nrf1 resembles Nrf2 but lacks Neh4 and the C-terminus of the Neh6 domain. Nrf proteins in ascidians and sea urchins were found to have a similar domain structure to Nrf1, including the NHB1 domain in their N-terminus. The Nrf proteins of fruit fly and octopus are also characterized by ER binding NHB1, but they lack Neh4 and Neh5 domains. Interestingly, the distance between the DLG and ETGE motifs in the Neh2 domain is highly conserved among Nrf2 proteins in vertebrates. For Nrf2 ubiquitination, these two motifs must be appropriately bound to Keap1 homodimer as a hinge and latch that bridges the two Keap1 molecules [97]; therefore, the distance between DLG and ETGE must be tightly controlled so that Nrf2 fits in the Keap1 homodimer structure. The distance between the two motifs was relatively short in ascidians and sea urchins and relatively long in octopus and fly, possibly suggesting that the binding structure of Keap1-Nrf2 varies among species.

Zebrafish have the second Nrf2 homolog, Nrf2b [103]. Although Nrf2b possesses Neh1, -2 and -3 domains in a conserved manner, it totally lacks the transcription activation motif in Neh4, and the homology of the phosphorylation site in Neh6 domain is low, implying that this homolog does not have a conserved function with mammal Nrf2. Timme-Laragy et al. [103] pointed out the possibility that Nrf2b mainly represses transcription, which corresponds to the loss of important domains in this co-ortholog.

### 4.2. Possible Evolutionary Path of Nrf Proteins

**Figure 4:**
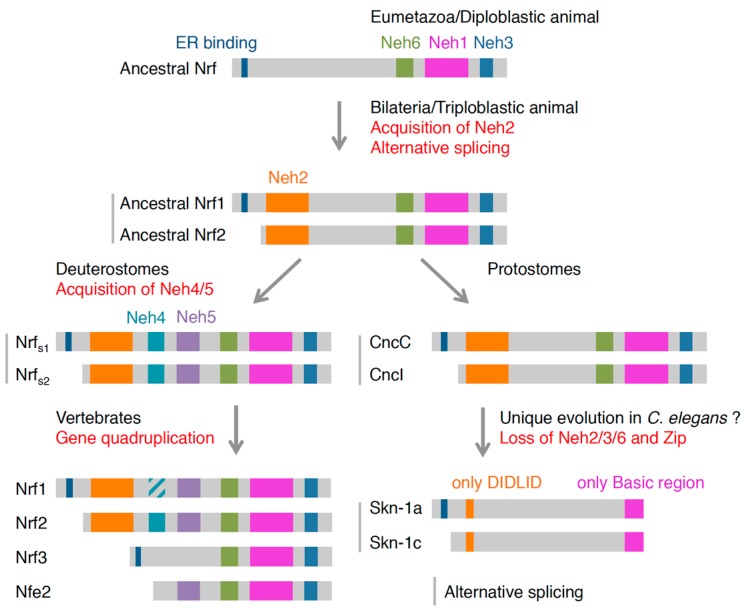
The evolution of Nrf/Cnc transcription factors deduced from amino acid sequences. Gray bars and subscripts (s1 and s2) in Deuterostomes Nrf denote products of alternative splicing.

From the structural analysis above, we deduced the evolutionary history of the Nrf proteins (Figure 4). Of the six domains of Nrf2 in mammals, ancestral Nrf might possess Neh1, -3 and -6 as well as the ER binding region in the N-terminus. As the *Hydra* Nrf protein lacks an Neh2 domain and possesses an ER binding domain, its activity is suspected to be regulated mainly at the binding to the ER membrane, just like Nrf1 in mammals. The rise of the Neh2 domain in triploblasts may correspond to the appearance of Keap1-dependent regulation and probably activation in response to various stressors. Data for sea urchins and ascidians have suggested that deuterostomes obtained Neh4 and -5 domains, while protostomes (fly and octopus) did not. *C*. *elegans* evolved a unique protein, Skn-1, that lacks most of the original domains, even the C-terminal half of Neh1 (leucine zipper).

At the appearance of vertebrates, the Nrf gene locus was diversified into four loci, which vary in domain structure. In addition to obtaining six complete functional Neh domains, Nrf2 lost the ER binding domain, thus suggesting that Nrf2 had avoided localization to the ER. Recent advances have shown that *Drosophila* generates at least 16 variants of Cnc, one of which (CncI) has a similar structure to Nrf2 that lacks the N-terminal ER binding domain [5]. In addition, information from the octopus database has shown that two mRNA variants are generated from the same Nrf locus, one of which encodes the protein with the ER binding domain and the other lacking this domain. Skn-1 was also shown to have splicing variants with a membrane binding domain (Skn-1a) and without it (Skn-1c). The localization of Skn-1a to the ER was also reported [104]. Furthermore, the genomic information of *Ciona intestinalis* shows that the Nrf locus generates two variants: one with an ER binding domain and the other without it.

Taken together, these findings suggest that, at the latest, the mechanism of alternative splicing and generation of varied proteins from a single Nrf locus was already present at the appearance of triploblastic animals. In vertebrates, at gene quadruplication, varied proteins are assigned to each replicated locus. For the Nrf2 locus, a transcription factor regulated by a Keap1-dependent and ER-independent mechanism is assigned.

## 5. Evolution of Keap1

### 5.1. Comparison of Keap1 Proteins

We performed a similar phylogenetic analysis of Keap1 (Table 2). In zebrafish, two Keap1 co-orthologs (Keap1a and Keap1b) have been found [105]. Interestingly, teleosts, including amazon molly, cavefish, cod, fugu, medaka, platyfish, spotted gar, stickleback, tetraodon and tilapia, all have two Keap1 co-orthologs (http:www.ensembl.org). To determine the evolutionary origin of this duplication, we further consulted the genomic information of the African coelacanth (*Latimeria chalumnae*) [106], green anole lizard (*Anolis carolinensis*) [107] and *Xenopus tropicalis*. Two Keap1 co-orthologs were found in the coelacanth and frog but not in the lizard. The second Keap1 is suspected to have been generated at the appearance of vertebrates and lost in amniotes.

**Figure 5:**
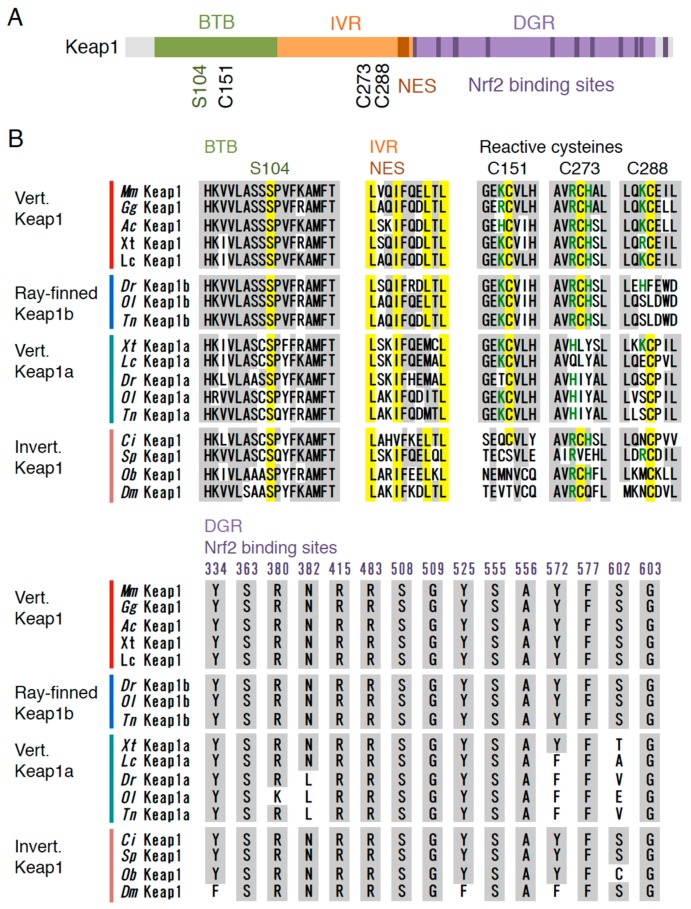
A comparison of Keap1 proteins: (**A**) domain structures of Keap1; and (**B**) amino acid sequences of Keap1 proteins from mouse (*Mm*), chicken (*Gg*), anole lizard (*Ac*), clawed frog (*Xt*), coelacanth (*Lc*), zebrafish (*Dr*), medaka (*Ol*), green spotted puffer (*Tn*), ascidian (*Ci*), sea urchin (*Sp*), octopus (*Ob*) and fruit fly (*Dm*). The amino acids identical to mouse Nrf2 are shaded in gray. The serine residues essential for homodimer formation in the BTB domain, NES consensus sequence in the IVR domain and three reactive cysteine residues are shaded in yellow.

Keap1 is composed of three domains, broad complex-tramtrack-bric a brac (BTB), intervening region (IVR) and double glycine repeat (DGR) domains (Figure 5A), all of which are important for the inhibition of Nrf2 activity. N-terminal BTB is the essential region for the formation of the homodimer of Keap1. Without this dimerization, Keap1 is unable to ubiquitinate Nrf2, and Ser-104 in this domain is reported to be necessary for dimer formation [108]. Of note, this serine, including the surrounding amino acids, is highly conserved in both vertebrates and invertebrates (Figure 5B).

The C-terminal DGR domain is the Nrf2 binding region. A detailed structural analysis revealed the direct binding sites in Keap1 to the DLG and ETGE motifs of Nrf2 [97]. Nrf2 binding to this domain is competitively inhibited by proteins that have ETGE-like motifs such as p62 and partner and localizer of BRCA2 (PALB2) [30,109,110], suggesting that the interface between the DGR and Neh2 domains functions as a sensor for a certain type of cellular stress including autophagy deficiency and DNA damage. Although some amino acids in this Nrf2 binding surface in the DGR domain had been replaced with others with similar physicochemical traits, most of these sites were highly conserved from flies to mice. Keap1-Nrf2 interaction might therefore already have existed at the rise of the ancestor of these animals (Figure 5B). Furthermore, Ser-602 is not conserved in Keap1a of frogs and fish. We previously showed that both Keap1a and Keap1b in zebrafish were able to inhibit Nrf2 activity [105,111]. Ser-602 may not be important for binding with Nrf2. The IVR domain interacts with Cul3 protein, which comprises the E3 ligase complex together with Roc1 [41,112,113]. This domain has a consensus sequence of nuclear export signal (Lx_(1–3)_Lx_(2–4)_LxL), which is important for localization at the cytoplasm [114]. This signal sequence was also highly conserved among species, implying that there is conserved regulation of the intracellular localization of Keap1.

The notable characteristic of Keap1 is its richness in cysteine residues. Given their high reactivity, cysteines can function as “sensor” amino acids [115]. When cysteines are attacked by reactive chemicals, the function of Keap1 is hindered by structural changes, which results in Nrf2 activation [116,117,118]. Of the 25 cysteines in mouse Keap1, Cys-151, Cys-273 and Cys-288 were shown to have sensor functions in vivo [119,120,121]. Cys-273 and its surrounding region were found to be highly conserved among the animal species investigated, except for sea urchins. Cys-288 was also detected in all of the species investigated, suggesting that these two cysteines are the oldest cysteines that function in stress sensing. Of these two cysteines, Cys-273 has been lost from Keap1a in lower vertebrates, while Cys-288 has been lost from Keap1b in ray-finned fish (Actinopterygii). Cys-151 was found in Keap1 of ascidians or higher animals, while sea urchins, octopus and fruit flies had not conserved this cysteine. These differences provide important information for understanding the diversification of Keap1 proteins and their function.

**Figure 6:**
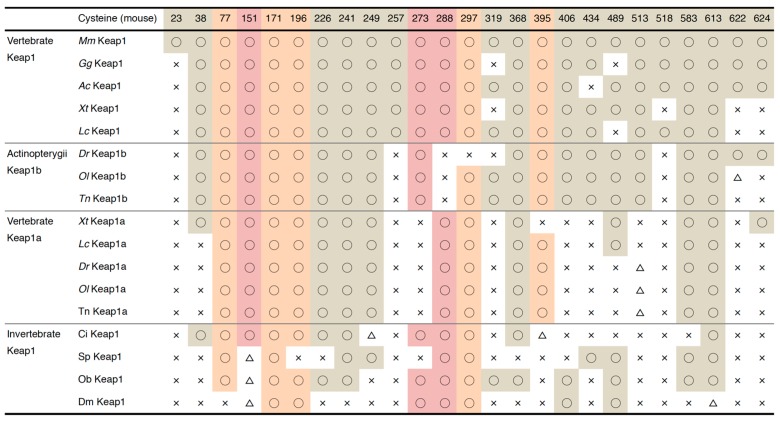
A summary of the cysteine residues of Keap1. The conservation of each cysteine is indicated as follows ○: conserved; △: not conserved but cysteine exists within three amino acids; ×: not conserved. Sensor cysteines are shaded in red, and cysteine residues conserved among Kelch family proteins in mice are shaded in orange.

Regarding other cysteines, as summarized in Figure 6, Cys-77, Cys-171, Cys-196, Cys-297 and Cys-395 are well-conserved among Kelch family proteins in mice; therefore, their evolutionary origin may be older than the appearance of Keap1. Eight cysteines (Cys-226, Cys-241, Cys-319, Cys-368, Cys-406, Cys-489, Cys-583, and Cys-613) are found in either flies or octopus, suggesting that these cysteines are conserved from ancestral Keap1. Other cysteines may also have been obtained over the course of evolution; for example, sea urchins have Cys-249 and Cys-434, and ascidians have Cys-38.

### 5.2. Possible Evolutionary Path of Keap1 Cysteines and Their Sensor Function

**Figure 7:**
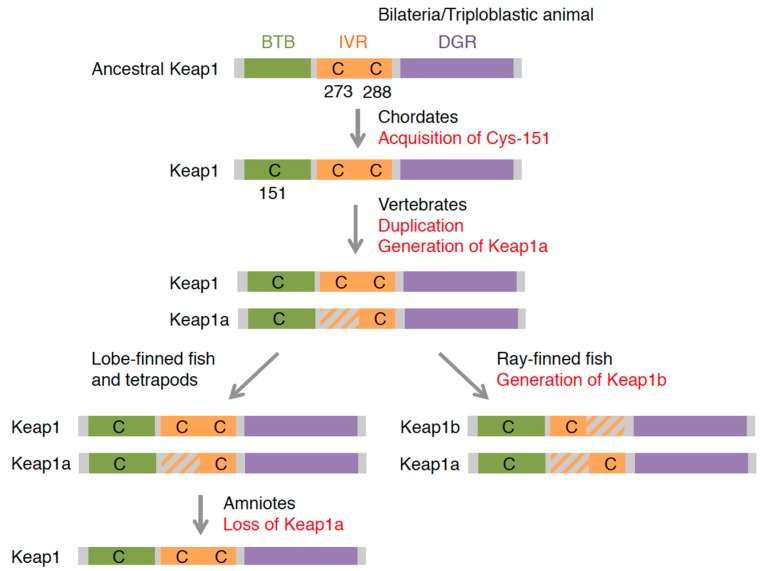
The evolution of Keap1 proteins deduced from amino acid sequences.

We attempted to clarify how Keap1 changed during evolution (Figure 7). Of the three well-studied “sensor” cysteines, ancestral Keap1 possessed only Cys-273 and Cys-288. Cys-151 was obtained at the appearance of chordates. In vertebrates, the Keap1 locus was duplicated, one of which lost Cys-273. Ray-finned fish further lost Cys-288 from another Keap1 co-ortholog, resulting in two distinct types of Keap1: Cys-273-lost (Keap1a) and Cys-288-lost (Keap1b) types. However, in lobe-finned fish (Sarcopterygii) and tetrapods, there was no change in these cysteines, and amniotes lost the C273-lost type Keap1, giving them only a single Keap1 locus.

Cys-151 has been determined to be an important cysteine for the detection of electrophiles, such as *tert*-butyl hydroquinone (tBHQ), sulforaphane, diethyl maleate (DEM) and dimethyl fumarate (DMF) [111,116,120,121]. Importantly, the sensor function of this cysteine is not determined only by Cys-151 itself, but also by the surrounding amino acid. Kobayashi et al. [111], from the functional difference of two zebrafish Keap1 co-orthologs, revealed that neighboring lysine, due to its positive charge, is essential for Cys-151 to function as the sensor (see Figure 5B). In ascidians, neighboring lysine is not conserved, so whether or not this cysteine has a sensor function is unclear. A possible evolutionary scenario is that Cys-151 was coincidentally obtained in chordates, and positive charged amino acids that appeared in vertebrates subsequently empowered the cysteine to be a sensor. Although fruit flies have not conserved this cysteine, the ARE reporter gene was activated by DEM treatment [5,122]. The mechanism for sensing electrophiles in flies is not fully elucidated, but other Keap1 cysteines may sense the signal, or a completely different mechanism may be involved. It was reported that nitric oxide, an endogenous gaseous transmitter, also targets Cys-151 and activates the Nrf2 system in mammals [121,123]. It will be interesting to examine whether or not an Nrf system with Keap1 that has no Cys-151 responds to this internal signal.

Cys-273 and Cys-288 have been shown to be essential for repressing Nrf2 activity under basal conditions [118,119]. However, the cysteines may not be important per se, since Keap1a and Keap1b in ray-finned fish lack Cys-273 and Cys-288, respectively, but both are able to repress Nrf2 [105,111]. Investigating the relationship between the structures around these cysteines and the Nrf2-repressing function of Keap1 will prove useful. These cysteines have been shown to be targeted by 15d-PGJ_2_, an anti-inflammatory prostaglandin [117,121]. We previously demonstrated that both co-orthologs in zebrafish, Keap1a and Keap1b, could function as sensors for 15d-PGJ_2_, implying that having either Cys-273 or Cys-288 is enough for 15d-PGJ_2_ sensing [111]. Acrolein and 4-hydroxynonenal also targets Cys-288 [123]. Although no evidence has yet shown that the Keap1-Cnc system in fruit flies responds to these chemicals, fly Keap1 probably senses this signal by its conserved Cys-273/288. Arsenic compounds potently induce Nrf2 and attack all three cysteines: Cys-151, Cys-273 and Cys-288 [121]. The responsiveness of the Keap1-Nrf2/Cnc system to arsenic is well-conserved in mammals [124], zebrafish [77] and flies [5,122], and all three of these species have at least one cysteine from among Cys-151, -273 or -288, which explains this conserved sensing mechanism reasonably well.

Recent progress has shown that Cys-226 and Cys-613 are necessary for the sensing of hydrogen peroxide and heavy metals such as zinc, cadmium, arsenic and selenium [123,125,126]. These cysteines are widely conserved among species, although sea urchin Keap1 does not have Cys-226, and fly Keap1 lacks both. In the zebrafish Keap1-Nrf2 system that respond to hydrogen peroxide and cadmium [76,111], these cysteines may function as sensors in a conserved manner. However, in fruit flies, although the Keap1-Cnc system responds to oxidative stressors, such as hydrogen peroxide and paraquat [58], Cys-226 and Cys-613 are not found in Keap1 (see Figure 6). This indicates that there must be different sensor mechanisms for oxidative stress and heavy metals in *Drosophila*.

In addition to the cysteines described above, Cys-257, Cys-297 and Cys-319 were determined to have highly reactive thiols [115,127]. Cys-257 was only possessed by Keap1 in coelacanths, frogs, lizards, chickens and mice, while Keap1a isoforms do not have this cysteine, suggesting that this cysteine evolved only in Keap1 after the appearance of lobe-finned fish (Figure 6). Although Cys-297 is one of the cysteines that are conserved among Kelch family proteins in mice, it was found to be highly conserved among the species examined, except for Keap1b of zebrafish (Figure 6). The presence of Cys-319 varied among species, and its evolutionary origin remains unclear. Cys-434 is modified by 8-nitro-cGMP, an endogenous electrophilic compound generated after nitric oxide production [128]. This cysteine is conserved in sea urchins and among vertebrates, except for anole lizards (Figure 6). The functions of these cysteine have not be determined biologically, but whether or not these chemicals activate Nrf, especially in species that do not have targeted cysteines in Keap1, will be of interest.

## 6. Future Directions

Similar to *Drosophila* Cnc and *C*. *elegans* Skn-1, which have been shown to be essential factors for embryonic development, vertebrate Nrf2 may perform functions other than those related to the stress response, even though Nrf2-knockout mice and Nrf2 mutant zebrafish develop normally [8,11,76]. Some evidence supports this notion, such as its known roles: first, Keap1-knockout mice, in which Nrf2 is constitutively active, developed hyperkeratosis in the esophagus and forestomach [129]. Second, Mitsuishi et al. showed that an active Nrf2 system in cancer cells upregulates the anabolic pathway through the transcriptional activation of pentose phosphate pathway enzymes, which enhances the cell proliferation [35]. We recently found that a gene encoding the pentose phosphate pathway enzyme phosphogluconate dehydrogenase (*pgd*) is also under the regulation of Nrf2 in zebrafish [80], suggesting that there is a conserved function among vertebrates. Zebrafish, in addition to mice, will prove a useful model for studying cell proliferation during normal development as well as under pathological conditions.

Growing evidence shows that endogenous stressors are involved in the pathogenicity of various diseases. Whether or not the interaction of the Keap1-Nrf system with the ER stress response and autophagy pathway are conserved in lower animals remains unclear; however, Skn-1 is known to have a close relationship with the ER stress response pathway [104]. Determining how the Keap1-Nrf system evolved its sensing mechanism against endogenous stressors will prove interesting. As we showed in this review, experimental evidence from lower model animals provides valuable information. Recently, the CRISPR-Cas9 technique has been more and more easily applied to non-model organisms [130,131], which will facilitate the accumulation of experimental evidence from various species. These data should be analyzed together with phylogenetic information, which will promote not only our understanding of the evolution of anti-stress mechanism, but also clarify its potential medical applications.

## Figures and Tables

**Table 1:** Summary of Nrf proteins.

Nrf2	*Mm* Nrf2	ENSMUSP00000099733
	*Gg* Nrf2	ENSGALP00000032649
	*Xt* Nrf2	ENSXETP00000003783
	*Dr* Nrf2a	ENSDARP00000062853
	*Dr* Nrf2b	ENSDARP00000106581
Nrf1	*Mm* Nrf1	ENSMUSP00000080467
	*Gg* Nrf1	ENSGALP00000035379
	*Xt* Nrf1	ENSXETP00000047513
	*Dr* Nrf1a	ENSDARP00000094757
	*Dr* Nrf1b	ENSDARP00000127352
Nrf3	*Mm* Nrf3	ENSMUSP00000005103
	*Xt* Nrf3	ENSXETP00000026569
	*Dr* Nrf3	ENSDARP00000015027
Nfe2	*Mm* Nfe2	ENSMUSP00000122476
	*Xt* Nfe2	ENSXETP00000057159
	*Dr* Nfe2	ENSDARP00000002745
Invertebrate Nrf	*Ci* Nrf	ENSCINP00000024999
	*Sp* Nrf	XP_011683763
	*Ob* Nrf	XP_014784776
	*Dm* CncC, CncI	NP_732833.1, NP_001247258.1
	*Hm* Nrf	XP_002160548.1
	*Ce* Skn-1a, Skn-1c	NP_741404.1, NP_741405

**Table 2:** Summary of Keap1 proteins.

Vertebrate Keap1	*Mm* Keap1	ENSMUSP00000131029
	*Gg* Keap1	ENSGALP00000046666
	*Ac* Keap1	ENSACAP00000008820
	*Xt* Keap1	ENSXETP00000063060
	*Lc* Keap1	ENSLACP00000008916
Keap1b	*Dr* Keap1b	ENSDARP00000124228
	*Ol* Keap1b	ENSORLP00000004762
	*Tn* Keap1b	ENSTNIP00000007190
Keap1a	*Xt* Keap1a	ENSXETP00000049635, Xenbase (http://www.xenbase.org/)
	*Lc* Keap1a	ENSLACP00000018705
	*Dr* Keap1a	ENSDARP00000045763
	*Ol* Keap1a	ENSORLP00000017543
	*Tn* Keap1a	ENSTNIP00000020338
Invertebrate Keap1	*Ci* Keap1	ENSCINP00000017048
	*Sp* Keap1	XP_003724241.1
	*Ob* Keap1	XP_014782077.1
	*Dm* Keap1	NP_788685.1
